# Surveillance of Antimalarial Resistance *Pfcrt*, *Pfmdr1*, and *Pfkelch13* Polymorphisms in African *Plasmodium falciparum* imported to Shandong Province, China

**DOI:** 10.1038/s41598-018-31207-w

**Published:** 2018-08-28

**Authors:** Chao Xu, Qingkuan Wei, Kun Yin, Hui Sun, Jin Li, Ting Xiao, Xiangli Kong, Yongbin Wang, Guihua Zhao, Song Zhu, Jingxuan Kou, Ge Yan, Bingcheng Huang

**Affiliations:** Shandong Institute of Parasitic Diseases, Shandong Academy of Medical Sciences, Shandong Provincial Reference Laboratory for Malaria Diagnosis, Jining, 272033 China

## Abstract

Antimalarial drug resistance is a major public health problem in China. From 2012 to 2015, more than 75% of malaria cases in Shandong Province were *P*. *falciparum* returned from Africa. However, molecular marker polymorphisms of drug resistance in imported *P*. *falciparum* cases have not been evaluated. In this study, we analyzed polymorphisms of the *Pfcrt*, *Pfmdr1*, and *Pfkelch13* genes in 282 *P*. *falciparum* cases returned from Africa to Shandong between 2012 and 2015. Among the isolates, polymorphisms were detected in codons 74–76 of *Pfcrt* and 86, 184, 1246 of *Pfmdr1*, among which K76T (36.6%) and Y184F (60.7%) were the most prevalent, respectively. Six *Pfcrt* haplotypes and 11 *Pfmdr1* haplotypes were identified and a comparison was made on the prevalence of haplotypes among East Africa, West Africa, Central Africa and South Africa. One synonymous and 9 nonsynonymous mutations in *Pfkelch13* were detected in the isolates (4.6%), among which a candidate artemisinin (ART) resistance mutation P553L was observed. The study establishes fundamental data for detection of chloroquine resistance (CQR) and ART resistance with molecular markers of the imported *P*. *falciparum* in China, and it also enriches the genetic data of antimalarial resistance for the malaria endemic countries in Africa.

## Introduction

Malaria is one of the most important parasitic diseases presented in many countries that causing a serious global public health problem. There were approximately 214 million malaria cases and 438,000 deaths worldwide in 2015, of which approximately 88% occurred in Africa^1^. *P*. *falciparum*, the most threatening among five species of malaria parasites associated with human infection, has been mainly responsible for the majority of the morbidity and mortality around the world^2^. During the past decades, drug resistance of *P*. *falciparum* has become a critical obstacle to control and eliminate malaria. Long-term anti-malarial monotherapy is capable of causing the emergence of drug resistant parasite strains and may provide a chance to spread the resistance to sensitive parasite population in other epidemic areas under conditions suitable *Anopheles* mosquitoes for transmission^3^. Since the early 1940s, chloroquine (CQ) was continuously used for treatment of malaria in many countries, which has been confirmed one of the most important antimalarial drugs with quick metabolism, good curative effects, and low prices^4^. However, due to excessive drug usage over the years, the CQ resistance (CQR) of *P*. *falciparum* isolates was found initial emerging from Thailand-Cambodia border in Southeast Asia in 1957 and Venezuela-Colombia border in Northern South America in 1959^5,6^, and eventually spread to other countries around the world. In 2006, the World Health Organization (WHO) recommended artemisinin-based combination therapies (ACTs) as the first-line treatment for uncomplicated *P*. *falciparum*^7^. ACTs serve as an important therapeutic method to avoid or defer the development of drug resistance for *P*. *falciparum* infection. Unfortunately, *P*. *falciparum* resistance to artemisinin (ART), the cornerstone of ACTs, was emergence in western Cambodia and subsequently spreading across several neighboring countries in Greater Mekong Subregion (GMS) of Southeast Asia in recent years^8–13^. Moreover, the emergence of ART resistant indigenous isolates in Africa has been reported more recently, which would be anxious in the future since the suitable replacement drugs are limited^14^. Thus, it is urgent to monitor the drug resistant trend of *P*. *falciparum*, so as to assess the possibility of reintroducing conventional drugs and also attempt to block the emergence of potential large-scale ART resistant transmission.

Molecular marker detection for parasite’s drug resistance is one of several methods for the surveillance of resistant prevalence and antimalarial efficacy^15^. The single nucleotide polymorphisms (SNPs) at codons 72, 74, 75 and 76 of *P*. *falciparum* chloroquine resistance transporter gene (*Pfcrt*), and 86, 184, 1034, 1042, and 1246 of *P*. *falciparum* multidrug resistance 1 gene (*Pfmdr1*) have been shown to be associated with parasite’s CQR^16,17^. Moreover, *Pfkelch13*, a gene locating on chromosome 13 of *P*. *falciparum* and encoding K13-propeller protein, was identified playing a vital role in conferring ART resistance through whole genome sequencing of ART resistance isolate and vitro ring-stage survival assays (RSA_0–3h_)^18^. To date, 13 nonsynonymous mutations (P441L, F446I, G449A, N458Y, Y493H, R539T, I543T, P553L, R561H, V568G, P574L, C580Y, A675V) on *Pfkelch13* gene of *P*. *falciparum* have been reported to be associated with clinical ART resistance that occurred in Southeast Asia^19^. Among them, Y493H, R539T, I543T, and C580Y were validated ART-resistant mutations, whereas the rest were candidate resistance mutations^20^.

In China, owing to substantial efforts for strategies and intervention over the past decades, the country has achieved a great success in controlling malaria transmission, with morbidity and mortality dramatically reduced to low levels^21^. In 2010, the Chinese Government initiated the National Malaria Elimination Program (NMEP), in order to eliminate malaria nationwide by the year 2020^22^. Currently, indigenous malaria parasite was almost absented in majority of regions in China other than some local transmission still occurred in Yunnan Province and Tibet Autonomous Region^23^. However, due to the intensive commercial intercourse, travelling and migrant laborers, a markedly rise of imported cases in recent years has posed a severe threat to eradicate malaria^24^. There has been no indigenous malaria patient reported in Shandong Province of China since 2012, whereas the imported cases have increased gradually, especially *P*. *falciparum* coming back from Africa was predominant^25^. Since little is known about the molecular basis of drug resistance of imported *P*. *falciparum* in Shandong Province, we investigated polymorphisms and haplotypes distribution of *Pfcrt* and *Pfmdr1*, and mutations in *Pfkelch13* of *P*. *falciparum* isolates returned from Africa in Shandong between 2012 and 2015, in order to accumulate and update baseline data for molecular surveillance linked to antimalarial drug resistance in China.

## Results

### Epidemiologic profile of cases

Totally 282 uncomplicated *P*. *falciparum* cases that returned from 23 countries of Africa to Shandong Province from 2012 to 2015 were enrolled in this study. Among these cases, the majority was coming back from Central Africa (35.1%, 99/282), followed by Southern Africa (30.5%, 86/282), Western Africa (26.6%, 75/282), Eastern Africa (7.8%, 22/282), but no patient was returned from Northern Africa (Table 1). There were 279 male patients and 3 female patients (93:1). The distribution of patients by age ranged from 19 to 60, with 92.6% (261/282) occurred from 20 to 50 years old. The patient’s occupations were mainly consisted of farmers (58.2%, 164/282) and laborers (23.1%, 65/282), and also included business (8.9%, 25/282), house workers (6.0%, 17/282), sailors (1.4%, 4/282), and other unknown occupations (2.5%, 7/282). Before travelling to Africa, all patients received health education conducted by local CDCs, including knowledge about malaria transmission and diagnosis, personal protection, mosquito prevention, and standard treatments. The patients accepted ACTs treatment specified according to the guidelines and regimens for the use of antimalarial drugs in China (2009)^26^. Clinical features showed that all patients recovered well after they took the therapy and there is no malaria recrudescence through continuous follow-up.

**Table 1:** Imported cases returned from regions and countries of Africa.

Regions and countries	No. of cases
***Eastern Africa***	**22**
Sudan	12
Tanzania	8
Ethiopia	2
***Western Africa***	**75**
Nigeria	34
Ghana	20
Guinea	11
Liberia	3
Sierra Leone	2
Niger	2
Ivory Coast	1
Burkina Faso	1
Mali	1
***Central Africa***	**99**
Equatorial Guinea	77
Republic of Congo	12
Cameroon	6
Chad	3
Gabon	1
***Southern Africa***	**86**
Angola	66
Mozambique	13
Zambia	4
Malawi	1
Madagascar	1
South Africa	1
**Total**	**282**

### Polymorphisms of *Pfcrt* and *Pfmdr1* genes

Among the 282 isolates, 279 (98.9%) for *Pfcrt* gene and 272 (96.5%) for *Pfmdr1* gene were successfully sequenced after PCR amplification, whereas 3 isolates of *Pfcrt* (Equatorial Guinea: n = 2, Nigeria: n = 1) and 10 isolates of *Pfmdr1* (Equatorial Guinea: n = 2, Ghana: n = 2, Nigeria: n = 1, Sudan: n = 1, Ethiopia: n = 1, Niger: n = 1) were failed to obtain sequences due to poor quality of DNA. The prevalence of *Pfcrt* and *Pfmdr1* polymorphisms were shown in Table 2. The entire codons 72–76 of *Pfcrt* were well examined and no polymorphism was found in the position 72 and 73. Of the codons 74, 75 and 76, 36.6% (102/279) of isolates carried polymorphisms and K76T accounting for the same percentage was the most prevalent (36.6%, 102/279). For *Pfmdr1*, the residues 130, 1034, 1042 and 1109 of all isolates were wild-type alleles. Among the mutant alleles at codons 86, 184 and 1246, polymorphisms were found in 65.4% (178/272) isolates and Y184F with 60.7% (165/272) prevalence was more frequent than the others. In addition, 0.7% (2/272) of the isolates carried D1246Y mutation.

**Table 2:** Polymorphisms of *Pfcrt* and *Pfmdr1* in isolates returned from Africa.

Gene	Codons position	No. of isolates	Prevalence of mutation
*Pfcrt* (N = 279)	M74I	100	35.8%
	N75E/D	100	35.8%
	K76T	102	36.6%
*Pfmdr1* (N = 272)	N86Y	82	30.2%
	Y184F	165	60.7%
	D1246Y	2	0.7%

### Geographic distribution of *Pfcrt* haplotypes

**Figure 1:**
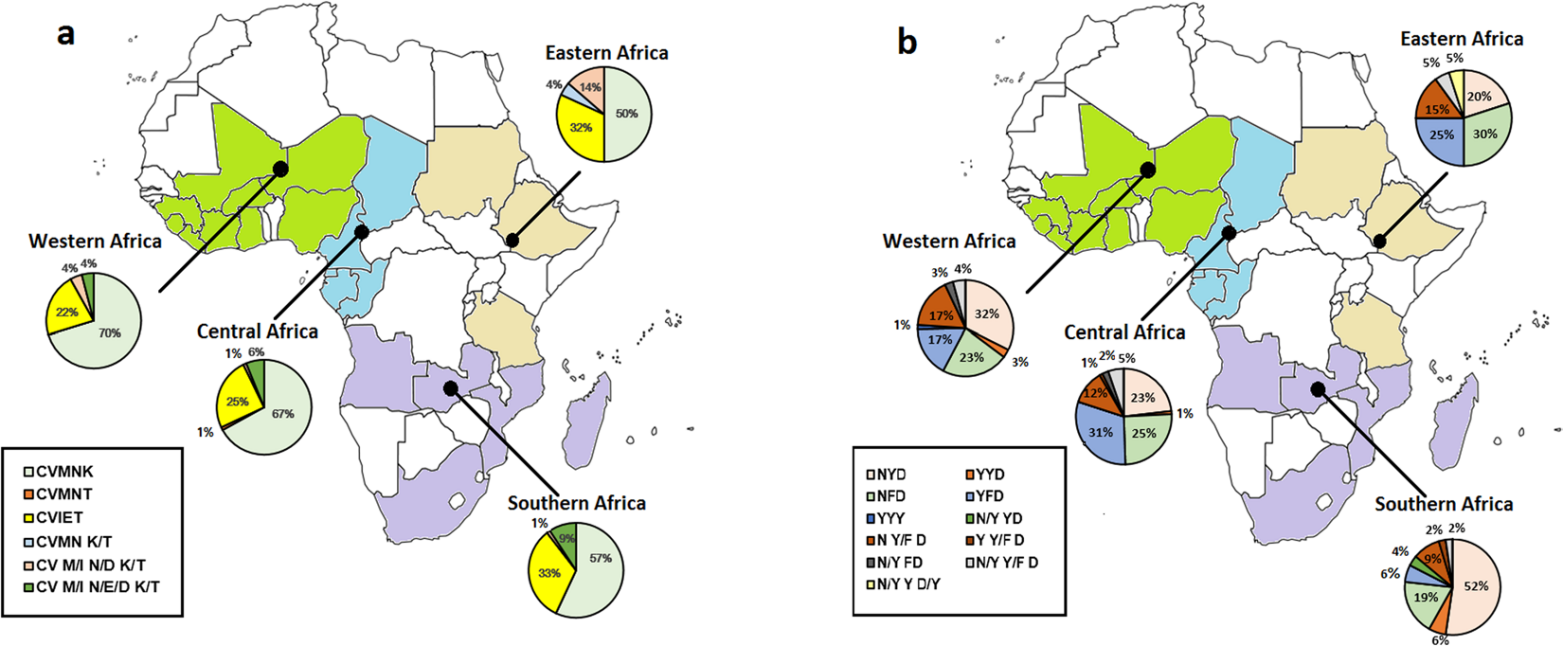
Geographical distribution of *Pfcrt* (Panel a) haplotypes and *Pfmdr1* (Panel b) haplotypes in imported *P*. *falciparum* isolates from Africa. Color difference in the map represents the parasites distribution in Africa. No isolate involved in the study are shown in white. The isolates returned from Eastern Africa, Western Africa, Central Africa and Southern Africa are shown in primrose yellow, green, light blue and purple, respectively. Pie charts presenting the frequencies of different haplotypes.

Of the *Pfcrt* gene, 6 haplotypes coding 72–76 were confirmed, including wild type C_72_V_73_M_74_N_75_K_76_ (CVMNK), mutant type CVMNT (mutated amino acids underlined), CVIET, and mixed type CVMN K/T, CV M/I N/D K/T, CV M/I N/E/D K/T, with prevalence of 63.4% (177/279), 0.4% (1/279), 26.9% (75/279), 0.4% (1/279), 2.9% (8/279) and 6.1% (17/279), respectively. Of these, CVMNT and CVMN K/T were only detected once in the isolates. Among the other four haplotypes, CVMNK, CVIET and CV M/I N/D K/T were found in all regions from Africa with no isolate carried CV M/I N/E/D K/T from Eastern Africa. The prevalence of CVMNK in Eastern Africa, Western Africa, Central Africa, and Southern Africa was 50.0% (11/22), 70.3% (52/74), 67.0% (65/97), and 57.0% (49/86), respectively. There was no significant difference among the groups (χ^2^ = 5.28, *P* > 0.05). The largest proportion of isolates with CVIET was returned from Southern Africa (32.6%, 28/86), followed by Eastern Africa (31.8%, 7/22), Central Africa (24.7%, 24/97) and Western Africa (21.6%, 16/74), and no significant difference was observed (χ^2^ = 2.95, *P* > 0.05). The total proportion of mixed type haplotypes (CVMN K/T, CV M/I N/D K/T, and CV M/I N/E/D K/T) was most occurred in Eastern Africa (18.2%, 4/22), followed by Southern Africa (10.5%, 9/86), Western Africa (8.1%, 6/74), and Central Africa (7.2%, 7/97), and there was no significant difference among the groups (χ^2^ = 2.81, *P* > 0.05). The detail information about distribution of *Pfcrt* haplotypes is shown in Table 3 and Fig. 1a.

**Table 3:** Distribution of *Pfcrt* and *Pfmdr1* haplotypes in isolates returned from Africa.

Gene	Haplotype	Eastern Africa	Western Africa	Central Africa	Southern Africa	Total
*Pfcrt*	CVMNK	11 (50.0%)	52 (70.3%)	65 (67.0%)	49 (57.0%)	177 (63.4%)
	CVMNT	0	0	1 (1.0%)	0	1 (0.4%)
	CVIET	7 (31.8%)	16 (21.6%)	24 (24.7%)	28 (32.6%)	75 (26.9%)
	CVMN K/T	1 (4.5%)	0	0	0	1 (0.4%)
	CV M/I N/D K/T	3 (13.6%)	3 (4.1%)	1 (1.0%)	1 (1.2%)	8 (2.9%)
	CV M/I N/E/D K/T	0	3 (4.1%)	6 (6.2%)	8 (9.3%)	17 (6.1%)
	Total	22	74	97	86	279
*Pfmdr1*	NYD	4 (20.0%)	23 (32.4%)	22 (23.2%)	45 (52.3%)	94 (34.6%)
	YYD	0	2 (2.8%)	1 (1.1%)	5 (5.8%)	8 (2.9%)
	NFD	6 (30.0%)	16 (22.5%)	24 (25.3%)	16 (18.6%)	62 (22.8%)
	YFD	5 (25.0%)	12 (16.9%)	29 (30.5%)	5 (5.8%)	51 (18.8%)
	YYY	0	1 (1.4%)	0	0	1 (0.4%)
	N/Y YD	0	0	0	3 (3.5%)	3 (1.1%)
	N Y/F D	3 (15.0%)	12 (16.9%)	11 (11.6%)	8 (9.3%)	34 (12.5%)
	Y Y/F D	0	0	1 (1.1%)	2 (2.3%)	3 (1.1%)
	N/Y FD	0	2 (2.8%)	2 (2.1%)	0	4 (1.5%)
	N/Y Y/F D	1 (5.0%)	3 (4.2%)	5 (5.3%)	2 (2.3%)	11 (4.0%)
	N/Y Y D/Y	1 (5.0%)	0	0	0	1 (0.4%)
	Total	20	71	95	86	272

### Geographic distribution of *Pfmdr1* haplotypes

Totally, 11 haplotypes were identified according to variation of codons 86, 184, and 1246, including wild type N_86_Y_184_D_1246_ (NYD), mutational-types YYD, NFD, YFD, YYY, and mixed type N/Y YD, N Y/F D, Y Y/F D, N/Y FD, N/Y Y/F D, N/Y Y D/Y, accounting for 34.6% (94/272), 2.9% (8/272), 22.8% (62/272), 18.8% (51/272), 0.4% (1/272), 1.1% (3/272), 12.5% (34/272), 1.1% (3/272), 1.5% (4/272), 4.0% (11/272) and 0.4% (1/272), respectively. NFD, YFD and N Y/F D were the top three prevalent of the non-wild haplotypes. The highest percentage of the NYD haplotype was found in Southern Africa, accounting for 52.3% (45/86), followed by Western Africa (32.4%, 23/71), Central Africa (23.2%, 22/95), and Eastern Africa (20.0%, 4/20). There was significant difference among the groups (χ^2^ = 19.49, *P* < 0.05). Among the Eastern, Western, Central and Southern areas, the overall mutational type haplotypes accounted for 55.0% (11/20), 43.7% (31/71), 56.8% (54/95) and 30.2% (26/86) respectively, and significant difference among the groups was observed (χ^2^ = 13.83, *P* < 0.05). The proportion of total mixed type haplotypes was 25.0% (5/20) in Eastern Africa, 23.9% (17/71) in Western Africa, 20.0% (19/95) in Central Africa, and 17.4% (15/86) in Southern Africa, and there was no significant difference among the groups (χ^2^ = 1.27, *P* > 0.05). The detail information about distribution of *Pfmdr1* haplotypes is shown in Table 3 and Fig. 1b.

### Analysis of *Pfkelch13* mutations

**Figure 2:**
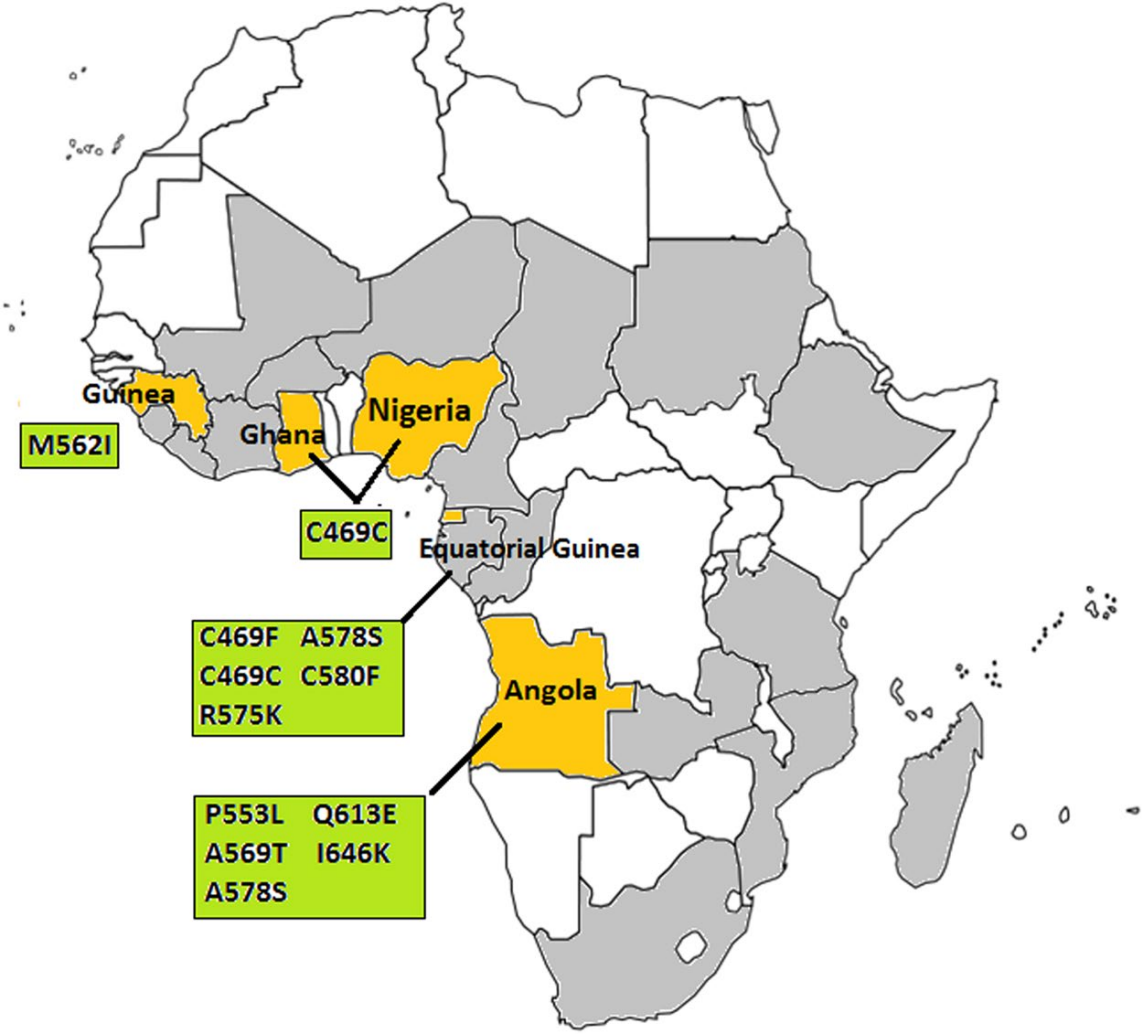
Geographical distribution of *Pfkelch13* mutations in imported *P*. *falciparum* isolates from Africa. Color difference in the map represents the parasites distribution in Africa. No isolates involved in the study are shown in white. The isolates with no *Pfkelch13* mutations are shown in gray. The isolates carried *Pfkelch13* mutations are shown in yellow. The *Pfkelch13* mutations are shown in green box.

Propeller domain of the *P*. *falciparum Pfkelch13* gene was successful sequenced from all 282 samples and no isolate carried more than one *Pfkelch13* mutation. The distribution of *Pfkelch13* mutations is shown in Fig. 2. The prevalence of *Pfkelch13* mutations was 4.6% (13/282), among which isolates from Equatorial Guinea (1.8%, 5/282) and Angola (1.8%, 5/282) were more frequent than others. Ten different mutant alleles including one synonymous and 9 nonsynonymous were observed, of which C469C, M562I and I646K were unreported before, and C469F and R575K were not previously reported in African isolates (Table 4). Among them, C469C was the most prevalent synonymous mutation (1.1%, 3/282) and A578S was the most prevalent nonsynonymous mutation (0.7%, 2/282), whereas the rest were observed only once. Notably, a candidate resistance mutation P553L was observed in an isolate back from Angola. However, none of the validated ART resistant mutations were observed in the parasites returned from Africa.

**Table 4:** *Pfkelch13* mutations in isolates returned from Africa. S, synonymous mutation. NS, nonsynonymous mutations.

Mutation	Type	Source countries (No. of isolates)
C469F	NS	Equatorial Guinea (N = 1)
C469C*	S	Equatorial Guinea (N = 1), Ghana N = 1), Nigeria (N = 1)
P553L	NS	Angola (N = 1)
M562I*	NS	Guinea (N = 1)
A569T	NS	Angola (N = 1)
R575K	NS	Equatorial Guinea (N = 1)
A578S	NS	Equatorial Guinea (N = 1), Angola (N = 1)
C580F	NS	Equatorial Guinea (N = 1)
Q613E	NS	Angola (N = 1)
I646K*	NS	Angola (N = 1)

*Mutation was unreported before.

## Discussion

In response to uncomplicated *P*. *falciparum* infections, ACTs including oral compound tablets of dihydroartemisinin plus piperaquine (DHA + PQ), artesunate plus amodiaquine (AS + AQ), and ART + PQ were routine regimen for the current treatment in China^27^. The widespread resistance to CQ, AQ, and the emergence of ART resistant isolates in Africa could attract China’s attention since the resistant parasites would enter the country with increasing migrants. Therefore, understanding the molecular mutation profiles and geographical distribution of drug resistance of *P*. *falciparum* is urgent and important for the effective treatment of malaria in China.

Shandong Province is an important coastal economic region located in Eastern China. Historically, Shandong was one of the most severe malaria transmission areas in China, with millions of annual malaria case numbers outbreak in the province during the 1960s and 1970s^28^. Relying on substantial efforts for anti-malarial campaigns over the decades, the malaria cases in the province decreased sharply and epidemics was well controlled. Nevertheless, there was an increasing trend of the malaria cases presented in Shandong from 2012 to 2015 due to the proportion of imported malaria (100%, 586/586) and the majority of them were *P*. *falciparum* cases returning from African countries (79.9%, 468/586)^29,30^. Since 2009, DHA + PQ and AS + AQ have been applied to against uncomplicated *P*. *falciparum* in Shandong as recommended by WHO in China^7,26^. Nevertheless, little is known about current drug resistance of imported malaria cases. Thus, we assessed polymorphisms of the *Pfcrt*, *Pfmdr1*, and *Pfkelch13* genes in order to provide genetic data for antimalarial drug resistance.

Polymorphisms in the amino acid positions 72–76 of *Pfcrt* gene are reliable markers for CQR of *P*. *falciparum* parasites, of which K76T mutation is the predominant^31^. In our study, *Pfcrt* mutant allele was found together with K76T mutation, which was consistent with above conclusion. For CQR parasites, CVIET and SVMNT are the two main mutant haplotypes prevalent worldwide^32,33^. The CVIET haplotype has been shown to be predominant in many African countries and is almost the unique haplotype with high frequency in some areas of Africa^34^. In this study, CVIET (26.9%) was more frequently than other mutant haplotypes and had no significant difference among four regions of Africa, was consistent with above conclusions. Interestingly, CVMNT haplotype was detected in an isolate returned from Equatorial Guinea in this study, which was also observed in 7% of Nigerian isolates and 70.6% of Ghananian isolates previously^35,36^, suggesting a distinct difference was present in epidemic distribution of CVMNT in Africa. Except mutant types, mixed genotypes of *Pfcrt* had been detected in 6.6% isolates from Equatorial Guinea^37^, and then 4.8% mixed types were found in parasites returned from ten countries in Africa^38^. In present study, 9.3% (26/279) isolates with *Pfcrt* mixed genotypes were found coming back from 11 source countries in Africa. It further enriched the geographical range of *Pfcrt* mixed types and also suggested *Pfcrt* mixed alleles were widely prevalent in African countries. It was known that the isolates with SVMNT haplotype were found most prevalent in South America and Southeast Asia but considered rare in Africa^39^. This was consistent with our results showing that no isolate carried SVMNT from Africa. However, several recent studies indicated that SVMNT had been detected in parasite strains from Tanzania and Angola. This could be associated with relatively low efficacy of AQ monotherapy in the countries^40,41^. Therefore, continuous surveillance of SVMNT haplotype is still required for African imported malaria in China. The cessation of CQ for a period of time may lead to the restoration of CQ sensitive parasites. In Malawi, the prevalence of CQR *Pfcrt* genotype decreased from 85% to 13% during 10 years after withdrawal of CQ^42^. The same situation also happened to patients from Southern Ethiopia and travelers returned from West and Central African countries^43,44^. In our samples, the parasites returned from Western Africa and Central Africa carried 70.3% and 67.0% of wild CVMNK haplotype in contrast to 21.6% and 24.7% of mutant CVIET haplotype respectively, which was consistent with above conclusion.

SNPs of *Pfmdr1* gene was selected for CQR, and they also had been reported to be associated with regulating drug susceptibility or tolerance to several antimalarials, for example, quinine (QN), mefloquine (MQ), lumefantrine (LU), and even ART^45^. In this study, *Pfmdr1* allelic variants was only observed in codons 86, 184 and 1246. Previous studies suggested that *Pfmdr1* N86Y mutation was a potential marker for CQR while Y184F may also play a role in mediating resistance to several antimalarial drugs^3,46^. Among the isolates, a high frequency of N86Y (30.2%) and Y184F (60.7%) were observed in our study, of which Y184F was more prevalent. It was similar to the previous results in Senegal (14.9% and 71.8%) and Equatorial Guinea (50.3% and 87.3%)^37,47^. In addition, linkage disequilibrium between K76T and N86Y has been observed in Africa previously^48^. In this study, both *Pfcrt* K76T and *Pfmdr1* N86Y were detected in 8.9% (25/282) parasite isolates. The correlation of two mutations associated with drug resistance will be of concern in further survey. Considering *Pfmdr1* codons 1034, 1042, and 1246, the mutational haplotypes has occurred frequently among CQR parasites in South America, whereas wild haplotype is common in CQR isolates from Africa and Asia^35^. Interestingly, D1246Y mutation were found in our samples, one reason for this might be ascribed to population flows between Africa and America. For *Pfmdr1* wild haplotypes and mutant haplotypes, frequency diversity was observed among four regions of Africa (*P* < 0.05) respectively. It might be related to diversity of drug pressure and transmission intensity among the countries in Africa.

ACTs are the first-line treatment for *P*. *falciparum* in the majority of endemic countries and has been identified as the most successful antimalarial drug over the past 10 years^49^. *Pfkelch13* gene is essential for molecular surveillance of malaria parasites with ART resistance. So far, more than 150 nonsynonymous mutations contracted in *Pfkelch13* gene have been reported^19^. In this study, 9 nonsynonymous *Pfkelch13* mutations were observed in the isolates and most of which were returned from Equatorial Guinea and Angola. One possible reason was that samples from two countries were significantly more than others. Previous study reported that R539T mutation was detected in isolates returned from Angola and Equatorial Guinea respectively^50^. In addition, R539T and C580Y was found in migrant workers returning from Ghana to China^36^. In our study, no validated ART-resistant *Pfkelch13* mutations was observed, the difference could be probably explained by sample sizes. Especially, a candidate ART-resistant mutation P553L was found in an isolate returned from Angola in this study, which also had been detected in Cambodia, Vietnam and West Africa^19^. These available data suggested that the extensive distribution of low frequent *Pfkelch13* nonsynonymous mutations in African *P*. *falciparum* population and the emergence of *Pfkelch13* mutations in Africa might lead the risk of global resistance transmission. The A578S mutation, which was commonly observed in Africa and several Southeast Asian countries, has been proven to be not related to clinical ART resistance^19^. In our study, A578S was the most prevalent nonsynonymous mutations among the samples, which was consistent with above results. Notably, one recent report showed a Chinese patient in Jiangsu Province carried ART resistant *P*. *falciparum* from Equatorial Guinea, indicating the emergence of indigenous ART resistant isolate in Africa^14^. Although there is no evidence that ART resistant *P*. *falciparum* parasite has emerged in Shandong, the attention should be paid to the increased imported malaria in the province. Therefore, routine molecular surveillance, clinical investigation and field research should be continuously strengthened for awareness of potential emergence of resistance to ACTs from Africa.

In conclusion, our study evaluated polymorphisms and geographic distribution of haplotypes of *Pfcrt* gene and *Pfmdr1* gene in uncomplicated *P*. *falciparum* cases imported from Africa to Shandong Province of China. The prevalence of *Pfcrt* K76T and *Pfmdr1* N86Y were still modestly present, indicating the presence of CQR in imported *P*. *falciparum* cases. We also detected one synonymous and 9 nonsynonymous mutations in propeller domain of *Pfkelch13* gene, among which a candidate ART resistance mutation P553L was observed and 3 mutations were unreported before. Nevertheless, no validated ART resistance mutation of *Pfkelch13* gene was found in this study, suggesting no immediate risk to the effect of ART. The study establishes fundamental data for the detection of CQR and ART resistance with molecular markers of the imported *P*. *falciparum* in China, and it also enriches the genetic data of drug resistance for the malaria endemic countries in Africa.

## Methods

### Sample and demographic data collection

Blood samples were obtained from malaria cases who returned from Africa to Shandong Province between 2012 and 2015 prior to antimalarial drug treatment. Demographic data of all cases were recorded, including gender, age, occupation and source countries. The confirmed diagnosis of *P*. *falciparum* was performed by microscopic examination of Giemsa-stained thick smears and nested PCR amplifying small-subunit rRNA gene of *Plasmodium* spp., as described previously^51,52^. For each specimen, approximately 200 µl finger-prick blood was spotted onto a piece of 3 MM Whatman filter paper. After air dried, blood papers were marked with names, serial numbers and dates, and then stored at −20 °C in individual pouch until DNA extraction.

### Ethical approval

This study was reviewed and approved by the Ethics Committee of Shandong Institute of Parasitic Diseases, Shandong Academy of Medical Sciences (Jining, China). All methods were performed in accordance with the relevant guidelines and regulations. The informed consent was obtained from all individual patients or their legal guardians prior to the research.

### DNA preparation

Parasitic DNA was isolated from filter paper blots through a QIAamp DNA mini kit (Qiagen, Valencia, CA, USA) according to the manufacturer’s instructions. The DNA template was kept at −20 °C until use.

### Nested PCR amplification

The known polymorphisms relating to drug resistance at codons 72, 74, 75, 76 of *Pfcrt* gene and codons 86, 130, 184, 1034, 1042, 1109, 1246 of *Pfmdr1* gene, and also mutations on propeller domain of *Pfkelch13* gene, were evaluated by nested PCR as described in previous studies^18,53,54^. The target fragments covering polymorphic sites were as follows: amino acid position 51–83 (a 145 bp portion) for *Pfcrt*, amino acid position 69–228 (a 526 bp portion) and 1030–1282 (a 799 bp portion) for *Pfmdr1*, and amino acid position 433–702 (a 849 bp portion) for *Pfkelch13*. The details of nested PCR primers and conditions are shown in the Supplementary Table S1. The products were analyzed by 1.5% agarose gel electrophoresis stained with SYBR Gold and visualized using a ChemiDoc XRS system (Bio−Rad, Hercules, CA, USA).

### DNA sequencing

The successful amplified PCR products were sequenced by the BGI Corporation (Beijing, China). Direct sequencing was carried out through a bigdye terminator v3.1 cycle sequencing kit and ABI prism 3730xl DNA analyzer (Applied Biosystems, Foster City, CA, USA) according to the manufacturer’s protocol. The sequences were evaluated by Blast search program on NCBI (https://blast.ncbi.nlm.nih.gov/Blast.cgi) to ensure accuracy of PCR amplicons. SNPs of sample sequences were analyzed in Bioedit 7.0 by comparing with reference 3D7 strain PF3D7_0709000 (*Pfcrt*), PF3D7_0523000 (*Pfmdr1*) and PF3D7_1343700 (*Pfkelch13*) from PlasmoDB (http://plasmodb.org/plasmo/). The mixed alleles were determined according to the emergence of two chromatogram peaks at one nucleotide site through the Mutation Surveyor v4.0.0 (SoftGenetics LLC., State College, PA, USA).

### Statistical analyses

Data was established using Microsoft Excel 2007 and analyzed by SPSS 19.0 (SPSS Inc., Chicago, IL, USA). The Chi squared test was used to evaluate differences among the groups. A *P*−value < 0.05 was considered to be statistical significance. The map was created by MapInfo 15.0 (Pitney Bowes, Troy, NY).

## Electronic supplementary material

Supplementary Information

## Data Availability

All data generated or analyzed during this study are included in this published article.
