# Supplementary material for: Surveillance of Antimalarial Resistance Pfcrt, Pfmdr1, and Pfkelch13 Polymorphisms in African Plasmodium falciparum imported to Shandong Province, China
Source: Sci Rep. 2018 Aug 28;8:12951. doi: 10.1038/s41598-018-31207-w (PMC6113250; doi:10.1038/s41598-018-31207-w)
Supplement: Supplementary file 1 — Supplementary Information [file 41598_2018_31207_MOESM1_ESM.docx]

Surveillance of Antimalarial Resistance *Pfcrt*, *Pfmdr1*, and *Pfkelch13* Polymorphisms in African *Plasmodium falciparum* imported to Shandong Province, China

# Chao Xu^+^, Qingkuan Wei^+^, Kun Yin, Hui Sun, Jin Li, Ting Xiao, Xiangli Kong, Yongbin Wang, Guihua Zhao, Song Zhu, Jingxuan Kou, Ge Yan & Bingcheng Huang*

Shandong Institute of Parasitic Diseases, Shandong Academy of Medical Sciences, Shandong Provincial Reference Laboratory for Malaria Diagnosis, Jining 272033, China

**^+^**These authors contributed equally to this work.

***Corresponding Author**

Prof. Bingcheng Huang

No.11 Middle Taibai Road, Jining, Shandong Province, 272033, People’s Republic of China.

E-mail: hbc863@hotmail.com

**Supplementary Information**

| **Gene** | **Primer** | **Sequence (5′-3′)** | **Product size (bp)** | **PCR condition** |
| --- | --- | --- | --- | --- |
| *Pfcrt* | CRT -1F | CCGTTAATAATAAATACACGCAG | 537 | 95 °C 5 min; followed by 30 cycles (92 °C 30 s, 56 °C 30 s, 60 °C 1 min); 60 °C 3 min; store 12 °C. |
|  | CRT-1R | CGGATGTTACAAAACTATAGTTACC |  |  |
|  | CRT-2F | TGTGCTCATGTGTTTAAACTT | 145 | 95 °C 5 min; followed by 30 cycles (92 °C 30 s, 48 °C 30 s, 65 °C 30 s); 60 °C 3 min; store 12 °C. |
|  | CRT-2R | CAAAACTATAGTTACCAATTTTG |  |  |
| *Pfmdr1* | MDR1-1F | TTAAATGTTTACCTGCACAACATAGAAAATT | 612 | 95 °C 3 min; followed by 35 cycles (93 °C 30 s, 52 °C 30 s, 72 °C 1 min); 72 °C 5 min; store 12 °C. |
|  | MDR1-1R | CTCCACAATAACTTGCAACAGTTCTTA |  |  |
|  | MDR1-2F | TGTATGTGCTGTATTATCAGGA | 526 | 95 °C 3 min; followed by 35 cycles (93 °C 30 s, 52 °C 30 s, 72 °C 1 min); 72 °C 5 min; store 12 °C. |
|  | MDR1-2R | CTCTTCTATAATGGACATGGTA |  |  |
|  | MDR2-1F | AATTTGATAGAAAAAGCTATTGATTATAA | 880 | 95 °C 3 min; followed by 35 cycles (93 °C 30 s, 52 °C 30 s, 72 °C 1 min); 72 °C 5 min; store 12 °C. |
|  | MDR2-1R | TATTTGGTAATGATTCGATAAATTCATC |  |  |
|  | MDR2-2F | GAATTATTGTAAATGCAGCTTTA | 799 | 95 °C 3 min; followed by 35 cycles (93 °C 30 s, 52 °C 30 s, 72 °C 1 min); 72 °C 5 min; store 12 °C. |
|  | MDR2-2R | GCAGCAAACTTACTAACACG |  |  |
| *Pfkelch13* | K13-1F | GGGAATCTGGTGGTAACAGC | 2063 | 94 °C 5 min; followed by 40 cycles (94 °C 30 s, 60 °C 1.5 min, 72 °C 1.5 min); 72 °C 10 min; store 12 °C. |
|  | K13-1R | CGGAGTGACCAAATCTGGGA |  |  |
|  | K13-2F | GCCTTGTTGAAAGAAGCAGA | 799 | 94 °C 5 min; followed by 40 cycles (94 °C 30 s, 60 °C 1.5 min, 72 °C 1.5 min); 72 °C 10 min; store 12 °C. |
|  | K13-2R | GCCAAGCTGCCATTCATTTG |  |  |

**Table S1: Primer sequences and nested PCR amplification conditions for *Pfcrt*, *Pfmdr1*, and *Pfkelch13* gene in *P. falciparum*.**
